# Mind bomb 2 limits inflammatory dermatitis in *Sharpin* mutant mice independently of cell death

**DOI:** 10.1093/pnasnexus/pgad438

**Published:** 2023-12-18

**Authors:** Daniel S Simpson, Holly Anderton, Jumana Yousef, Vineet Vaibhav, Simon A Cobbold, Esther Bandala-Sanchez, Andrew J Kueh, Laura F Dagley, Marco J Herold, John Silke, James E Vince, Rebecca Feltham

**Affiliations:** The Walter and Eliza Hall Institute of Medical Research, Parkville, Melbourne, VIC 3052, Australia; Department of Medical Biology, University of Melbourne, Parkville, Melbourne, VIC 3050, Australia; The Walter and Eliza Hall Institute of Medical Research, Parkville, Melbourne, VIC 3052, Australia; Department of Medical Biology, University of Melbourne, Parkville, Melbourne, VIC 3050, Australia; The Walter and Eliza Hall Institute of Medical Research, Parkville, Melbourne, VIC 3052, Australia; Department of Medical Biology, University of Melbourne, Parkville, Melbourne, VIC 3050, Australia; The Walter and Eliza Hall Institute of Medical Research, Parkville, Melbourne, VIC 3052, Australia; Department of Medical Biology, University of Melbourne, Parkville, Melbourne, VIC 3050, Australia; The Walter and Eliza Hall Institute of Medical Research, Parkville, Melbourne, VIC 3052, Australia; Department of Medical Biology, University of Melbourne, Parkville, Melbourne, VIC 3050, Australia; The Walter and Eliza Hall Institute of Medical Research, Parkville, Melbourne, VIC 3052, Australia; Department of Medical Biology, University of Melbourne, Parkville, Melbourne, VIC 3050, Australia; The Walter and Eliza Hall Institute of Medical Research, Parkville, Melbourne, VIC 3052, Australia; Department of Medical Biology, University of Melbourne, Parkville, Melbourne, VIC 3050, Australia; Olivia Newton-John Cancer and Wellness Centre, Austin Health, Melbourne, VIC 3084, Australia; School of Cancer Medicine, La Trobe University, Heidelberg, VIC 3084, Australia; The Walter and Eliza Hall Institute of Medical Research, Parkville, Melbourne, VIC 3052, Australia; Department of Medical Biology, University of Melbourne, Parkville, Melbourne, VIC 3050, Australia; The Walter and Eliza Hall Institute of Medical Research, Parkville, Melbourne, VIC 3052, Australia; Department of Medical Biology, University of Melbourne, Parkville, Melbourne, VIC 3050, Australia; Olivia Newton-John Cancer and Wellness Centre, Austin Health, Melbourne, VIC 3084, Australia; School of Cancer Medicine, La Trobe University, Heidelberg, VIC 3084, Australia; The Walter and Eliza Hall Institute of Medical Research, Parkville, Melbourne, VIC 3052, Australia; Department of Medical Biology, University of Melbourne, Parkville, Melbourne, VIC 3050, Australia; The Walter and Eliza Hall Institute of Medical Research, Parkville, Melbourne, VIC 3052, Australia; Department of Medical Biology, University of Melbourne, Parkville, Melbourne, VIC 3050, Australia; The Walter and Eliza Hall Institute of Medical Research, Parkville, Melbourne, VIC 3052, Australia; Department of Medical Biology, University of Melbourne, Parkville, Melbourne, VIC 3050, Australia

**Keywords:** mind bomb 2, Sharpin, cpdm, dermatitis, TNF

## Abstract

Skin inflammation is a complex process implicated in various dermatological disorders. The chronic proliferative dermatitis (cpd) phenotype driven by the cpd mutation (cpdm) in the Sharpin gene is characterized by dermal inflammation and epidermal abnormalities. Tumour necrosis factor (TNF) and caspase-8-driven cell death causes the pathogenesis of *Sharpin^cpdm^* mice; however, the role of mind bomb 2 (MIB2), a pro-survival E3 ubiquitin ligase involved in TNF signaling, in skin inflammation remains unknown. Here, we demonstrate that MIB2 antagonizes inflammatory dermatitis in the context of the cpd mutation. Surprisingly, the role of MIB2 in limiting skin inflammation is independent of its known pro-survival function and E3 ligase activity. Instead, MIB2 enhances the production of wound-healing molecules, granulocyte colony-stimulating factor, and Eotaxin, within the skin. This discovery advances our comprehension of inflammatory cytokines and chemokines associated with cpdm pathogenesis and highlights the significance of MIB2 in inflammatory skin disease that is independent of its ability to regulate TNF-induced cell death.

**Significance Statement**

The tightly controlled checkpoints on cell death and nuclear factor kappa B (NF-κB) signaling are crucial regulators of skin homeostasis. Mind bomb 2 (MIB2) is an inhibitor of cell death in cancer cells, but its role in other pathologies has not been explored. In these studies, we have used both targeted and unbiased approaches to interrogate the role of MIB2 in a mouse model of inflammatory skin disease. In contrast to its known pro-survival role in cancer, these approaches identified MIB2 as a promotor of the inflammatory wound-healing response *in vivo*. These findings enhance our understanding of cell death, specific inflammatory mediators, and their connection to dermatitis.

## Introduction

Skin inflammation is a complex pathological process involving the activation of immune cells, release of proinflammatory cytokines, and tissue damage. Dysregulation of this process can lead to dermatological disorders characterized by chronic inflammation, such as psoriasis and atopic dermatitis.

Sharpin (Src homology 3 [SH3] and multiple ankyrin repeat domains [SHANK]-associated Ras-related nuclear protein binding protein 2 [RANBP2]-type and C3HC4-type zinc finger containing 1 [RBCK1] homology [RH] domain-interacting protein), a constituent of the linear ubiquitin chain assembly complex (LUBAC), is a well-characterized regulator of skin homeostasis. In 1993, a spontaneous mutation arose in the *Sharpin* gene of C57BL/KaLawRij mice and was characterized by chronic proliferative dermatitis (cpd) (1). The cpd mutation (cpdm) results in a premature stop codon leading to loss-of-function of the Sharpin protein (2, 3). Notably, Sharpin deficiency reduces but does not abrogate, LUBAC activity, explaining the viability of *Sharpin* mutant animals when compared to the embryonic lethality resulting from loss of HOIL-1-Interacting protein (HOIP) or HOIL-1, which are both essential for LUBAC function (4–6).

*Sharpin^cpdm/cpdm^* (*Sharpin^cpdm^*) mice exhibit a severe psoriasis-like dermatitis phenotype that is characterized by dermal inflammatory infiltration and several epidermal abnormalities, including hyperkeratosis, acanthosis, and hyperplasia. The selective removal of *Sharpin* in keratinocytes recapitulates the inflammatory skin phenotype of *Sharpin^cpdm^* mice, indicating that expression of *Sharpin* in the epithelial keratinocytes is critical for preventing dermatitis (7). Further studies into the pathogenesis of the cpdm uncovered a range of additional multi-organ pathologies, including female infertility, a lack of Peyer's patches, epithelial orthokeratinization of the upper digestive tract, weight loss, perivascular inflammatory lesions of the lung and liver, abnormal, and elevated extramedullary haematopoiesis of the liver and spleen accompanied with splenomegaly and atrophy of the splenic white pulp (1, 8). Elevated cytokines and chemokines and eosinophilia are also associated with the tissue pathologies (8–10).

Excessive cell death contributes to the skin pathology observed in *Sharpin^cpdm^* mice. Cleaved caspase-8 and caspase-9 positive epidermal cells in *Sharpin^cpdm^* skin sections provide evidence that apoptotic pathways are associated with disease progression (11). Experiments with animal crosses found that the abrogation of tumour necrosis factor (TNF) or caspase-8 signaling, including keratinocyte-specific deletion of TNF receptor 1 (TNFR1), TNFR1 associated death domain (TRADD), or Fas associated via death domain (FADD), mitigated the skin pathology observed in *Sharpin^cpdm^* mice (12–14). These findings are consistent with the requirement of LUBAC for normal signaling to nuclear factor kappa B (NF-κB) and the prevention of cell death mediated by caspase-8 upon TNFR1 engagement (11, 12, 15).

Infiltrating immune cells were thought to be dispensable for the onset of dermatitis in *Sharpin^cpdm^* mice (1, 13, 16), however emerging evidence challenges this notion. Depletion of tissue-resident Langerhans cells (LCs) significantly reduces skin lesion development in *Sharpin^cpdm^* mice (17) and hematopoietic stem cell transfer (HSCT) experiments involving *Sharpin^cpdm^* bone marrow reveal that TNF production by LCs is crucial for the dermatitis phenotype (17). Moreover, conditional deletion of CYLD, a pro-death deubiquitinase, in myeloid mononuclear phagocytes completely rescues the *Sharpin^cpdm^* dermatitis phenotype (18). These findings indicate a pathological role for the immune compartment in *Sharpin^cpdm^* dermatitis.

MIB2 is an E3 ubiquitin ligase that plays a specific pro-survival role in TNF-driven apoptosis in cancer cells through the ubiquitin-mediated regulation of receptor-interacting protein kinase 1 (RIPK1), which limits TNFR1-driven activation of caspase-8 (19). MIB2 exhibits functional redundancy with other pro-survival regulators of TNF signaling. For example, depletion of MIB2 only sensitizes cells to TNF-induced cell death when TAK1 (transforming growth factor [TGF]-β-activated kinase 1) or the IAP (inhibitor of apoptosis) proteins are co-inhibited, or with co-treatment with cycloheximide, all of which compromise the survival signaling complex downstream of TNFR1 (19, 20).

Here we show that MIB2 is a novel regulator of skin inflammation in the context of the cpd mutation. Surprisingly, our data reveal that the regulatory function of MIB2 in skin inflammation is independent of its role in cell death or E3 ligase activity. Instead, we uncover a previously unrecognized role of MIB2 in the regulation of wound-healing-associated cytokines within the skin. The discovery of MIB2-mediated regulation of wound-healing cytokine production demonstrates the physiological role and importance of MIB2 in inflammatory-driven skin disease.

## Results

### MIB2 limits *Sharpin^cpdm^*-driven dermatitis

To assess the role of MIB2 and the E3 ligase activity of MIB2 in TNF-driven cell-death-dependent pathologies, we generated two strains of *Mib2* mutant C57BL/6J mice by CRISPR/Cas9. The first strain harbors a loss-of-function deletion mutation that spans exons 1–6 of the *Mib2* gene locus (*Mib2^−/−^).* The second strain harbors a missense F920A mutation in the 3′ end of the gene that reduces the E3 ubiquitin ligase activity of MIB2 (19). The F920A mutation is homologous to mutations in the X-linked inhibitor of apoptosis (XIAP) protein, which abolishes specific interactions between the phenylalanine residue and the hydrophobic isoleucine (Ile44) patch within ubiquitin thereby preventing ubiquitin transfer (21). The F920A knock-in (*Mib2^KI/KI^*) mice serve to test if any phenotypes observed in the *Mib2^−/−^* mice are specifically a consequence of defective MIB2 E3 ligase activity.

*Mib2^−/−^* and *Mib2^KI/KI^* animals reached weaning, aged well, and appeared overtly comparable to control animals (Fig. S1A). The preweaning mortality rate in both the *Mib2^−/−^* and *Mib2^KI/KI^* colonies was 36% and is average for other phenotypically normal colonies housed in our animal facility (Fig. S1B). Offsprings from heterozygous crosses were tested for Mendelian ratios to determine if *Mib2^−/−^* and *Mib2^KI/KI^* animals were comparably viable to control animals. In both the *Mib2^+/−^* by *Mib2^+/−^* and *Mib2^+/KI^* by *Mib2^+/KI^* crosses, all offspring conformed to the expected 1:2:1 ratio, indicating that deletion of *Mib2*, or the F920A mutation, does not impact murine viability (Fig. S1B).

To assess whether *Mib2^−/−^* or *Mib2^KI/KI^* animals exhibited any hematopoietic defects, peripheral blood was taken from age-matched wild-type control and *Mib2* mutant mice and analyzed for blood cell composition. ADVIA hematology analysis detailed a comparable percentage of hematocrit between genotypes that was made up of a similar number of reticulocytes and mature red blood cells (RBCs) (Fig. S1C). Platelet and white blood cell (WBC) counts between the genotypes were also similar (Fig. S1C). The total count and percentage composition of the leukocyte population including lymphocytes, neutrophils, monocytes, eosinophils, and basophils conformed to normal C57BL/6 peripheral blood profiles (Fig. S1C).

MIB2 expression has been reported in the mouse brain, heart, liver, and kidney (22); however, that analysis and most online databases utilize mRNA-based technologies to discern expression. To determine the expression of the MIB2 protein we performed a western blot analysis across a panel of tissues. MIB2 was specifically detected across all tissues examined except for the pancreas (Fig. S1D) and was also clearly expressed in bone marrow-derived macrophages (BMDMs), mouse dermal fibroblasts (MDFs), and murine epidermal keratinocytes (MEKs) (Fig. S1E). Interestingly, moderately elevated levels of MIB2 protein were present in *Mib2^KI/KI^* tissue relative to wild-type control animals, which is consistent with the really interesting new gene (RING) domain of MIB2 functioning, in part, to autoubiquitylate and degrade itself (Fig. S1F). Overall, these data demonstrated that MIB2 is widely expressed at the protein level in most murine tissues and in cell types commonly used for cell-death-based analyses.

To test whether MIB2 could limit TNF-driven cell-death-associated phenotypes *in vivo*, we crossed the *Mib2^−/−^* mice and *Mib2^KI/KI^* mice to *Sharpin^cpdm^* animals, which display numerous apoptotic and necroptotic pathologies (13). Notably, breeding pairs that contained a *Sharpin^cpdm/+^Mib2^−/−^* male and *Sharpin^cpdm/+^Mib2^−/−^* female were less successful than those with a *Sharpin^cpdm/+^Mib2^+/−^* or *Sharpin^cpdm/+^Mib2^+/+^* female (Fig. S2A). Similarly, *Sharpin^cpdm/+^Mib2^−/−^* male and female pairs exhibited a marked increase in the number of offspring dying before weaning, which can be prevented by a single wild-type copy of *Mib2* on either the paternal or maternal side (Fig. S2A). The dead offspring were either cannibalized or found dead, and of those that make it to weaning the expected genotypes are represented in Mendelian ratios (Fig. S2B). Overall, these data suggest that *Sharpin^cpdm/+^Mib2^−/−^* females that breed with *Sharpin^cpdm/+^Mib2^−/−^* males are potentially more stressed and neglect their offspring irrespective of the offspring genotype.

**Fig. 1.**
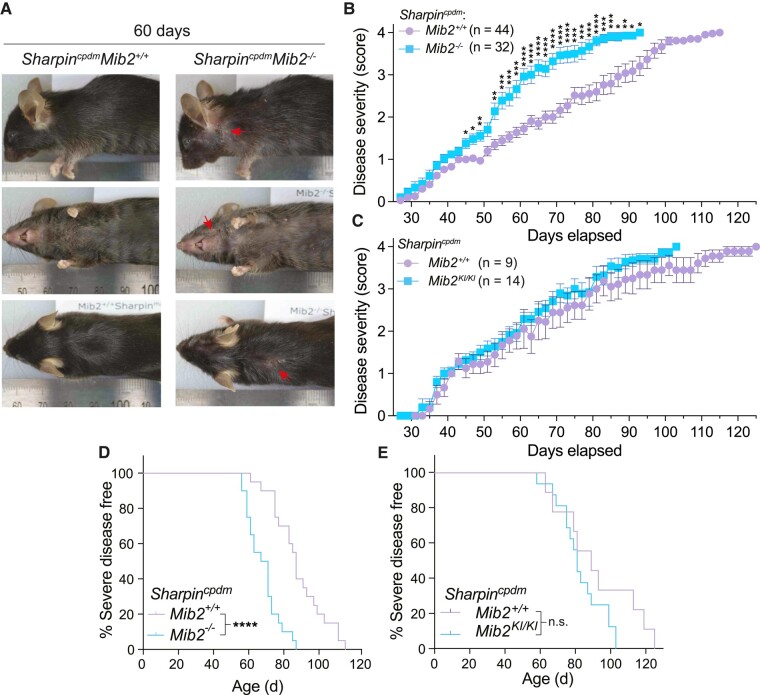
MIB2 limits *Sharpin^cpdm^*-driven dermatitis. A) Representative images of animals from the indicated genotypes at 60 days. Arrows indicate dermatitis. B) Dermatitis severity scoring for *Sharpin^cpdm^Mib2^−/−^* mice alongside cohoused cohorts of *Sharpin^cpdm^Mib2^+/+^* control animals. Severe disease is defined as reaching a clinical score of 4. Mean ± SEM is shown. *n* = 32–44. Significance determined by multiple Mann–Whitney *U* tests with a *Holm*–*Šídák* correction for multiple comparisons. Adj. *P*-value: <0.05 (*), ≤0.01 (**), ≤0.001 (***), ≤0.0001 (****). C) Dermatitis severity scoring for *Sharpin^cpdm^Mib2^KI/KI^* mice, controlled, and analyzed as in (B). *n* = 9–14. D) Severe-disease-free curves for animals in (B). Significance for disease-free curves was tested with a log-rank (Mantel–Cox) test. *P* ≤ 0.0001 (****). E) Severe-disease-free curves for animals in (C). Significance analyzed as in (D). n.s., not significant.

The cpd phenotype of the *Sharpin^cpdm^* animals is caused by a TNFR1-RIPK1/TRADD-caspase-8 cell death signaling axis, while most other organ phenotypes are necroptosis associated (13). We therefore hypothesized that the loss of MIB2 would exacerbate the cpd caused by the cpd mutation and have no effect on the other pathologies seen in these mice. Consistent with our hypothesis, deletion of MIB2 accelerated the progression of dermatitis caused by mutation of *Sharpin* when compared to a cohoused cohort of *Sharpin^cpdm^Mib2^+/+^* animals scored at the same time (Fig. 1A and B). However, reducing the E3 ubiquitin ligase activity of MIB2 with the F920A mutation did not impact dermatitis severity (Fig. 1C). Acceleration of disease progression resulted in a significantly reduced endpoint-based survival in *Sharpin^cpdm^Mib2^−/−^* mice (median survival = 69 days) compared to *Sharpin^cpdm^* control animals (median survival = 87 days; Fig. 1D). As expected, *Sharpin^cpdm^Mib2^KI/KI^* mice exhibited comparable endpoint-based survival to coscored *Sharpin^cpdm^* control animals (Fig. 1E). These findings show that MIB2 limits the skin phenotype of *Sharpin^cpdm^* mice, and that this likely occurs independently of the E3 ubiquitin ligase activity of MIB2.

**Fig. 2.**
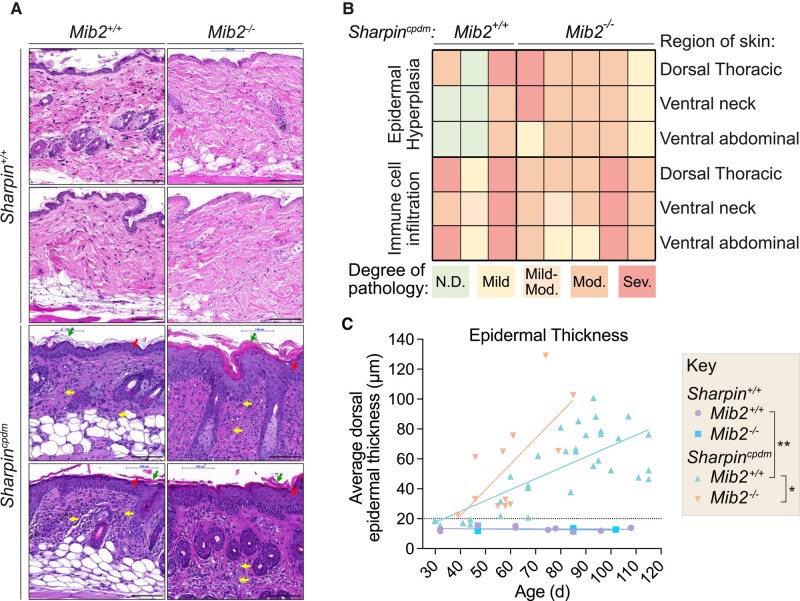
MIB2 restricts epidermal thickening during chronic proliferative dermatitis. A) H&E staining of skin sections from the dorsal thoracic region depicting epidermal hyperplasia and acanthosis (red arrows), parakeratotic hyperkeratosis (green arrows), and dermal immune cell infiltration (yellow arrows). *Sharpin^+/+^* sections are provided for reference. *n* = 13–33 (*Sharpin^cpdm^* animals) and 6–10 (*Sharpin^+/+^* controls). Images are representative of independent biological replicates. Scale bar = 100 μm. B) Histopathology analysis of skin sections from 56-day-old *Sharpin^cpdm^Mib2^−/−^* or *Sharpin^cpdm^Mib2^+/+^* control animals. mod., moderate; sev., severe; N.D., no pathology observed. Observations described as “numerous,” “marked,” or “extensive” are represented as severe. Each column represents an independent biological replicate, *n* = 3–5. C) Age-dependent quantification of epidermal thickness in mice from the indicated genotypes. Data points represent independent biological replicates, with a linear regression shown. *n* = 13–33 (*Sharpin^cpdm^* animals) and 6–10 (*Sharpin^+/+^* controls). Significance was determined by a linear regression test comparing the regression slopes. *P* < 0.05 (*), *P* ≤ 0.01 (**).

To identify whether the accelerated dermatitis in *Sharpin^cpdm^Mib2^−/−^* animals can be correlated with known histological pathologies reported to drive the cpdm phenotype, we assessed mice at ∼60 days (8–9 weeks) of age where *Sharpin^cpdm^Mib2^−/−^* animals are macroscopically more severe than *Sharpin^cpdm^* control animals (Fig. 1A and B). Histopathology analysis of skin sections validated that the histological features of the disease, epidermal hyperplasia, and acanthosis were more severe in *Sharpin^cpdm^Mib2^−/−^* mice (Fig. 2A). The severity of the epidermal hyperplasia in *Sharpin^cpdm^Mib2^−/−^* animals was significantly heightened across multiple dermal regions including the dorsal thoracic, ventral neck, and ventral abdominal regions when compared to *Sharpin^cpdm^* control and wild-type animals (Fig. 2B and C). Interestingly, *Sharpin^cpdm^Mib2^−/−^* mice displayed comparable parakeratotic hyperkeratosis and dermal immune cell infiltration to *Sharpin^cpdm^* control mice based on independent blinded assessments (Figs. 2A and S2C). Notably, there were no discernible differences in proliferation (Ki67), or populations of immune cells (CD45), lymphocytes (CD3), or F4/80^+^ cells between *Sharpin^cpdm^* control mice and *Sharpin^cpdm^Mib2^−/−^* mice (Fig. S2D).

### Absence of MIB2 does not influence skin-independent phenotypes in *Sharpin^cpdm^* animals

**Fig. 3.**
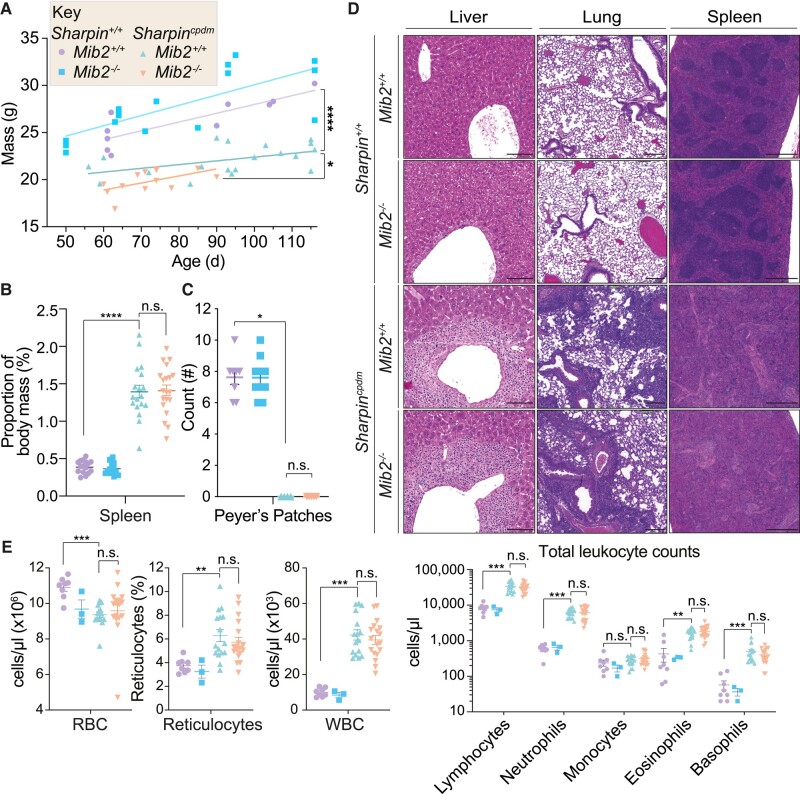
The absence of MIB2 does not influence the additional phenotypes present in *Sharpin^cpdm^* mice. A) Age-dependent quantification of body weight for the indicated genotypes. Data points represent independent biological replicates, with a linear regression shown. *n* = 10–19 biological replicates. Significance was determined by a linear regression test comparing the regression elevations. *P* < 0.05 (*), *P* ≤ 0.0001 (****). B) Quantification of spleen weights proportional to body mass for the indicated genotypes. Data points represent independent biological replicates. *n* = 15–20. Significance was determined using the Kruskal–Wallis test with Dunn's correction for multiple comparisons. Adj. *P*-value: ≥ 0.05 (n.s.), *P* ≤ 0.0001 (****). C) Quantification of Peyer's patches for the indicated genotypes. Data points represent independent biological replicates. *n* = 4–10. Significance was determined using the Kruskal–Wallis test with Dunn's correction for multiple comparisons. Adj. *P*-value: ≥0.05 (n.s.), <0.05 (*). D) H&E staining of liver, lung, and spleen sections from the indicated genotypes. *n* = 2–5. Images are representative of independent biological replicates. The indicated scale bars apply to each tissue group. Scale bar = 100 μm (liver), 200 μm (lung), and 500 μm (spleen). E) ADVIA quantification of whole blood from 9- to 18-week-old animals of red blood cell (RBC) and white blood cell (WBC) populations for the indicated genotypes. Data points represent independent biological replicates. *n* = 16–18 (*Sharpin^cpdm^* animals) and 3–8 (*Sharpin^+/+^* controls). Significance was determined using the Kruskal–Wallis test with Dunn's correction for multiple comparisons. Adj. *P*-value: ≥0.05 (n.s.), ≤0.01 (**), ≤0.001 (***).

*Sharpin^cpdm^* mice display several non-dermatological pathologies including weight loss, splenomegaly, and loss of Peyer's patches. Consistent with previous reports (8), *Sharpin^cpdm^* mice showed significantly lower weights compared to wild-type controls (Fig. 3A). A significant reduction in weight was also observed between *Sharpin^cpdm^* mice and *Sharpin^cpdm^Mib2^−/−^* mice (Fig. 3A), which was expected as the weight loss of these animals is linked to the severity of systemic inflammation and skin pathology (7). No significant differences were observed in *Sharpin^cpdm^*-driven splenomegaly or numbers of Peyer's patches between *Sharpin^cpdm^* mice and *Sharpin^cpdm^Mib2^−/−^* mice (Fig. 3B and C). Other histological pathologies of *Sharpin^cpdm^* mice, including immune cell infiltrate into the lung and liver, and atrophy of the splenic white pulp were also comparable between *Sharpin^cpdm^Mib2^−/−^* and *Sharpin^cpdm^* mice (Figs. 3D and S3A). Consistent with literature reports, peripheral blood from *Sharpin^cpdm^* mice also display elevated lymphocytes, granulocytes, and monocytes (13) (Figs. 3E and S3B). *Sharpin^cpdm^* and *Sharpin^cpdm^Mib2^−/−^* animals display a similar degree of granulocytosis (elevated neutrophils, eosinophils, and basophils) and lymphocytosis (elevated lymphocytes; Figs. 3E and S3B). Monocytosis of *Sharpin^cpdm^* mice was not observed in our cohorts (Fig. 3E), and although hematocrit was unchanged across all genotypes, *Sharpin* mutation did reduce total RBC counts and increase the percentage of reticulocytes within the RBC count, suggesting that loss of Sharpin may limit red blood cell maturation. These changes in the red blood cell compartment were, however, independent of *Mib2* status (Figs. 3E and S3B). Overall, our data suggest that the absence of MIB2 does not exacerbate the non-dermatological phenotypes observed in *Sharpin^cpdm^* mice.

### Loss of MIB2 does not enhance caspase-driven cell death

**Fig. 4.**
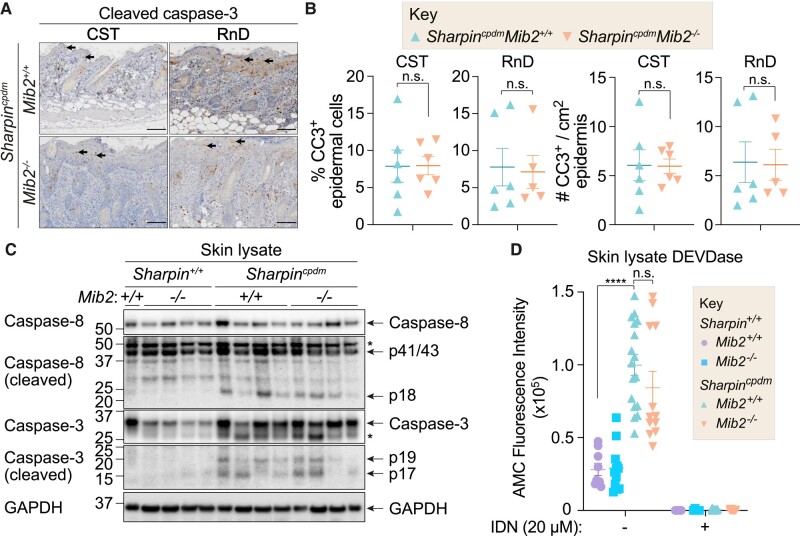
Loss of MIB2 does not enhance caspase-driven cell death. A) IHC of dorsal thoracic skin from 60-day-old *Sharpin^cpdm^Mib2^−/−^* and *Sharpin^cpdm^Mib2^+/+^* control animals depicting CC3 staining with either a cell signaling technologies antibody (CST) or RnD systems (R&D) antibody. Black arrows indicate representative CC3 +ve cells. One representative image is shown per group of *n* = 6 independent biological replicates. Scale bar = 100 μm. B) Quantification of CC3 positive epidermal cells as identified in (A). All comparisons were determined as nonsignificant (n.s.) by the Mann–Whitney *U* test. C) Western blot analysis of pro and cleaved caspased-3 and -8 from dorsal thoracic skin tissue lysate from the indicated genotypes. Each lane represents an independent biological replicate. *n* = 4. D) Caspase activity measured by DEVDase assay from dorsal thoracic skin tissue lysate for the indicated genotypes. Caspase inhibitor IDN-6556 (IDN) was used at 20 μM as a control for the assay postlysis. Data points represent independent biological replicates. *n* = 9–17. Significance was determined using the Kruskal–Wallis test with Dunn's correction for multiple comparisons. Adj. *P*-value: ≥ 0.05 (n.s.), ≤ 0.0001 (****).

To determine if the increased dermatitis of *Sharpin^cpdm^Mib2^−/−^* animals was associated with increased cell death, we performed cleaved caspase-3 (CC3) staining on thoracic dorsal skin, a region where the dermatitis phenotype consistently manifests, of age-matched 8- to 9-week-old *Sharpin^cpdm^Mib2^−/−^* and *Sharpin^cpdm^* mice (Fig. 4A). Keratinocytes within *Sharpin^cpdm^Mib2^−/−^* epidermal sections did not display a clear increase in CC3 staining compared with *Sharpin^cpdm^* control sections (Fig. 4A), and there was no significant differences in CC3 positive cells in the epidermis between the tested genotypes (Fig. 4B). Next, we analyzed cell death markers in whole skin lysates dissected from the dorsal thoracic region that the cpd typically presents in. CC3, cleaved caspase-8, and caspase activity similarly revealed no significant differences between *Sharpin^cpdm^Mib2^−/−^* and *Sharpin^cpdm^* animals (Fig. 4C and D). Given MIB2 is expressed in MDFs (Fig. S1E) we assessed whether loss of MIB2 sensitizes dermal fibroblasts to TNF-induced cell death. Treatment with TNF was effective in inducing cell death in *Sharpin^cpdm^* MDFs, and this cell death was partially attenuated by the RIPK1 kinase inhibitor Nec1s (Fig. S4A). Notably, the absence of MIB2 did not influence cell death under these treatment conditions, including cotreatment with a Smac mimetic/IAP antagonist (compound A) (23), caspase inhibitor (IDN-6556), or RIPK3 inhibitor (GSK’872) in combination with TNF (Fig. S4A and B). Western blot analysis of TNF-stimulated primary *Sharpin^cpdm^* and *Sharpin^cpdm^Mib2^−/−^* MDFs further showed that loss of MIB2 does not impact RIPK1 cleavage, CYLD phosphorylation, turnover or cleavage, or TNF-induced NF-κB activation and subsequent gene induction (Fig. S4C). The mild reduction in RIPK1 p-S166 observed (Fig. S4C) was not repeated in subsequent experiments. Collectively, these *ex vivo* findings indicate that MIB2 does not exert regulatory control over TNF-induced cell death in primary skin cell types within the framework of the cpd mutation.

### Loss of MIB2 leads to a significant reduction in wound-healing cytokines and chemokines

**Fig. 5.**
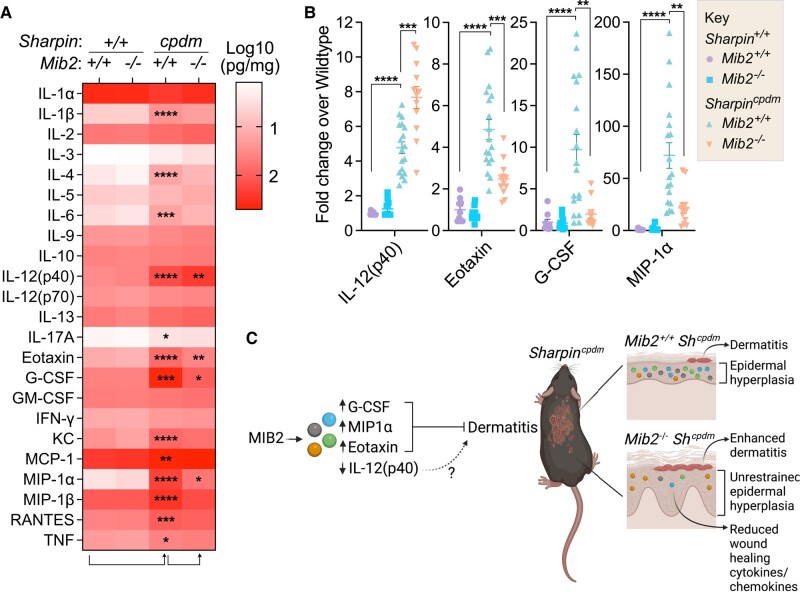
Loss of MIB2 leads to a significant reduction in wound-healing cytokines and chemokines. A) Heat map visualizing cytokine and chemokine concentrations (pg/mg skin tissue) measured by a multiplex assay of dorsal thoracic skin tissue lysate. *n* = 9–17. Significance between the indicated genotypes was determined multiple Mann–Whitney *U* tests with a *Holm*–*Šídák* correction for multiple comparisons. Significant comparisons are denoted as Adj. *P*-value: <0.05 (*), ≤0.01 (**), ≤0.001 (***), ≤0.0001 (****). B) Cytokine and chemokine concentrations (pg/mg skin tissue) measured by a multiplex assay of dorsal thoracic skin tissue lysate as in (A). Concentrations are displayed as a fold-change difference from the wild-type genotype. Data points represent independent biological replicates. *n* = 9–17. Significance between the indicated genotypes was determined multiple Mann–Whitney *U* tests with a *Holm*–*Šídák* correction for multiple comparisons. Significant comparisons are denoted as Adj. *P*-value: ≤0.01 (**), ≤0.001 (***), ≤ 0.0001 (****). C) Schematic depicting the regulation of *Sharpin^cpdm/cpdm^* (*Sharpin^cpdm^;Sh^cpdm^*)-driven dermatitis by MIB2. Loss of MIB2 leads to a reduction in MIP-1α, the critical wound-healing cytokines and chemokines G-CSF and Eotaxin, and an increase in IL-12(p40), unrestrained epidermal hyperplasia and enhanced *Sharpin^cpdm^*-driven dermatitis. Created with Biorender.com.

To assess alterations in inflammatory mediators at a systemic level, we conducted a Bio-Plex analysis of cytokines and chemokines in plasma samples collected from mice at 2 different stages of disease, 4-week-old mice and mice at the pathological endpoint (or at an equivalent age for the *Sharpin^+/+^* controls). Differences were observed in the plasma cytokine and chemokine profiles between *Sharpin^cpdm^* and *Sharpin^+/+^* controls; however, the deletion of *Mib2* did not significantly impact this inflammation irrespective of the cpd mutation (Fig. S5). Interestingly, striking and significant differences were observed in a number of cytokines and chemokines in skin homogenates from *Sharpin^cpdm^* and *Sharpin^cpdm^Mib2^−/−^* mice (Fig. 5A and B), with a notable reduction in macrophage inflammatory protein 1 alpha (MIP-1α), the key wound-healing molecules granulocyte colony-stimulating factor (G-CSF) and Eotaxin, and an increase in IL-12 (p40), in *Sharpin^cpdm^Mib2^−/−^* tissue (Fig. 5A and B). Importantly, these cytokines were not significantly changed in the *Mib2^−/−^* cohort and are exclusively associated with the cpd mutation (Fig. 5B). These findings reveal that MIB2 regulates the levels of specific wound-healing cytokines that may act to limit cpdm-driven dermatitis disease severity (Fig. 5C).

### Comparative mass spectrometry analysis of protein expression in skin lysates reveals differential protein expression in the absence of MIB2

**Fig. 6.**
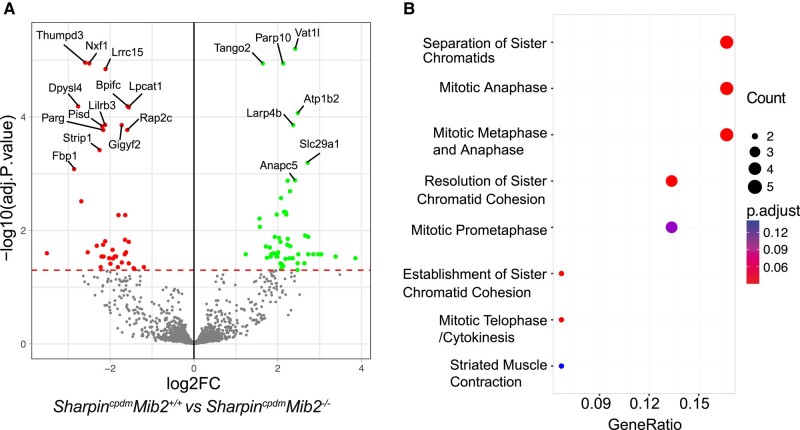
Comparative mass spectrometry analysis of protein expression in skin lysates reveals differential protein expression in the absence of MIB2. A) Volcano plot illustrating proteins exhibiting significant differential expression. The −log_10_ (BH corrected *P*-value) is plotted against the log_2_ protein fold changes comparing *Sharpin^cpdm^Mib2^+/+^* (red, left) vs. *Sharpin^cpdm^Mib2^−/−^* (green, right). Green = proteins upregulated in *Sharpin^cpdm^Mib2^−/−^* mice, and red = proteins upregulated in *Sharpin^cpdm^Mib2^+/+^* mice. Proteins were deemed differentially regulated with an adjusted *P*-value of ≤0.05 (proteins colored in red and green). B) Network plots of the reactome enrichment analysis using over-representation analysis (ORA) method for differentially expressed genes (DEGs) from *Sharpin^cpdm^Mib2^−/−^* vs. *Sharpin^cpdm^Mib2^+/+^* Illustrating the genes enriched in the mitotic anaphase pathway.

To identify how MIB2 might alter the proteomic landscape to limit progressive dermatitis, we performed an unbiased mass-spectrometry-based analysis of skin tissue lysates. Unique proteome characteristics associated with each genotype were observed in a principal component analysis (PCA) plot of the assessed *Sharpin^cpdm^Mib2^+/+^* and *Sharpin^cpdm^Mib2^−/−^*, *Mib2^−/−^* and wild-type mice, with several significant differentially enriched proteins identified in the *Sharpin^cpdm^Mib2^+/+^* vs. *Sharpin^cpdm^Mib2^−/−^*, *Mib2^−/−^* vs. wild-type, and *Sharpin^cpdm^ vs.* wild-type comparisons (Figs. 6A and S6A–C). A reactome enrichment analysis further revealed that proteins associated with cell division were more prevalent in the skin tissue of *Sharpin^cpdm^Mib2^−/−^* compared with *Sharpin^cpdm^Mib2^+/+^* mice (Fig. 6B), providing insights into the molecular mechanisms influenced by MIB2 and their potential impact on the disease. Collectively, our proteomic analysis unravels an intricate network of protein expression changes influenced by MIB2 deficiency in the context of cpdm-driven dermatitis.

## Discussion

MIB2 is a pro-survival E3 ubiquitin ligase that cooperates with the IAPs and TAK1 to limit the pro-apoptotic activities of RIPK1, thereby reducing TNF-mediated cancer cell death. In this study, we unveil a novel cell-death-independent function of MIB2 in the dermatological phenotype associated with the Sharpin cpd mutation. Our findings demonstrate that, in this context, MIB2 operates independently of its E3 ligase activity and pro-survival role, instead regulating distinct cytokines implicated in the wound-healing response.

Mice lacking MIB2 or carrying the F920A mutation, not in the context of the cpd mutation, exhibit a normal phenotype without any apparent pathological abnormalities. These observations show that MIB2 function is not essential for overall development, homeostasis, or the maintenance of basic physiological processes. The absence of overt phenotypic changes in these mice indicates that MIB2 may play a specific and context-dependent role in certain pathological conditions or disease contexts, such as the dermatological phenotype associated with the Sharpin cpd mutation we identify here. These findings emphasize the importance of studying MIB2 within specific genetic and environmental contexts to fully understand its functional significance and potential contributions to disease processes, which may identify potential therapeutic implications for targeting MIB2-regulated pathways.

The distinct skin phenotype, including exacerbated dermatitis and excessive epidermal hyperplasia observed in the absence of MIB2 in the context of the Sharpin cpd mutation is noteworthy, particularly given that no exacerbation of other cpdm-driven phenotype was observed. These additional phenotypes include the lack of Peyer's patches, weight loss, perivascular inflammatory lesions of the lung and liver, splenomegaly, and atrophy of the splenic white pulp. The absence of changes in these phenotypes suggests that the role of MIB2 in the development of these manifestations is minimal, indicating a tissue-specific function for MIB2. Interestingly, MIB2 may have disease-specific functions in regulating skin inflammation. Local depletion of the IAPs using a subcutaneous injection of the Smac-mimetic, compound A, induces inflammatory skin lesions driven by TNF and cell death. These lesions occur with rapid onset (1–3 days) relative to visible dermatitis in *Sharpin^cpdm^* animals and were not impacted upon deletion or inactivation of MIB2 (data not shown).

We found no evidence that MIB2 limits RIPK1 cytotoxicity and loss of MIB2 does not sensitize primary *Sharpin^cpdm^* fibroblasts to TNF-induced killing, even when the TNF signal is additionally perturbed with an IAP antagonist. Our whole tissue analysis and immunohistochemistry (IHC) examination of keratinocytes *in situ* also did not show an exacerbation of cell death in the absence of MIB2 at a timepoint where macroscopic clinical scores were observably different to *Sharpin^cpdm^* control animals. This indicates that augmented cell death in the absence of *Mib2* was not occurring in the skin of *Sharpin^cpdm^Mib2^−/−^* mice and, therefore, in this context MIB2 deletion does not restrain dermatitis in *Sharpin^cpdm^* animals by inhibiting TNF-induced and RIPK1-mediated cell death. The pro-survival role of MIB2 may be restricted to specific cell types, such as in cancer cells previously described (19), and/or specific circumstances. Future work to define the cell-death-associated functions of MIB2 *in vivo* will be of significant interest to explore.

Notably, the F920A mutation present in *Sharpin^cpdm^Mib2^KI/KI^* mice did not phenocopy MIB2 deletion. This mutation reduces but does not completely abrogate the E3 ubiquitin ligase activity of MIB2 (19). Therefore, the residual activity of the MIB2^F920A^ mutant might explain the wild-type-like behavior of the *Sharpin^cpdm^Mib2^KI/KI^* mice or, in our opinion, more likely indicates a ubiquitination-independent function of MIB2. One alternative mechanism of action may exist through MIB2 binding with RIPK1, an activity that the MIB2^F920A^ retains (19), yet if and how MIB2 binding to RIPK1 alters RIPK1 function in this context is unknown. Our findings do show though that MIB1 does not act redundantly with MIB2 in the context of the cpd mutation. This highlights the distinct roles of these closely related proteins, emphasizing the unique contribution of MIB2 in regulating skin inflammation-associated cpdm-linked dermatitis.

Additional processes contribute to the dermatitis caused by the cpd mutation of Sharpin, including TLR3 signaling (24), NLRP3 inflammasome and IL-1β signaling (25, 26), and IL-4 signaling (27). Alterations in other cytokines and chemokines are also evident in *Sharpin^cpdm^* mice; however, the interplay between these molecules, the immune response, and the dermatitis is poorly understood. Studies that performed hematopoietic stem cell transplantation experiments strongly suggest that the deletion of *Sharpin* in the immune cell infiltrate is dispensable for the inflammatory dermatitis phenotype (1, 13). Furthermore, transferring dermatitis affected *Sharpin^cpdm^* skin onto wild-type mice retains the dermatitis phenotype (16). These results suggest that the dermatitis phenotype of *Sharpin^cpdm^* animals is precipitated by keratinocyte death, while immune cells, such as TNF producing LCs (17) and myeloid cells expressing CYLD, drive the dermatitis phenotype (18).

MIP-1α and Eotaxin are chemokines that recruit macrophages and eosinophils to sites of injury. MIB2 is required for the efficient production of these chemokines in the cpd skin, but if or how changes in the levels of these molecules modulate dermatitis remains to be determined. The contribution of monocyte derived macrophages to dermatitis is not thoroughly explored in the *Sharpin^cpdm^* animals, although deletion of MIP-1α upon skin puncture demonstrates that this chemokine is dispensable for normal wound healing in this model (28). Moreover, recruitment of F4/80 positive cells to the skin of *Sharpin^cpdm^* animals was comparable in mice that had or did not have, MIB2, indicating no significant role for MIB2-mediated MIP-1α production in this aspect. On the other hand, administration of anti-IL-5 effectively depletes eosinophils and mildly increases the severity of epidermal thickness in *Sharpin^cpdm^* skin (29), providing some evidence that eosinophils limit dermatitis severity in this model. More broadly, eosinophils aid wound healing and epithelial remodeling (30) and the accelerated dermatitis we observe in the absence of MIB2 could be explained by the reduction in eotaxin and a subsequent defect in eosinophil recruitment.

Growth factor G-CSF is elevated in *Sharpin^cpdm^* skin, but its role in the skin pathology has remained unexplored. G-CSF enhances wound healing in animal excisional wound models (31) and in patients with dystophic epidermolysis bullosa or radiation-induced moist desquamation of the skin (32). Yet, G-CSF also promotes autoinflammatory skin pathology in phospholipase C gamma 2-associated antibody deficiency and immune dysregulation (APLAID) (33). Loss of MIB2 in the context of the cpd mutation reduced the abundance of G-CSF in the skin that correlated with enhanced dermatitis severity, suggesting G-CSF acts to promote wound healing in this inflammatory dermatitis model. Future genetic and biological (e.g. administration of G-CSF neutralizing antibody) experiments will help better define the function of G-CSF in the pathology of *Sharpin^cpdm^* skin.

A significant increase in IL-12(p40), a component of the bioactive IL-12 and IL-23 cytokines, was also observed in skin homogenates of *Sharpin^cpdm^Mib2^−/−^* mice compared to *Sharpin^cpdm^* mice. It is unlikely that MIB2 limits dermatitis by suppressing IL-12 signaling, as the administration of IL-12 resolves the dermatitis of *Sharpin^cpdm^* mice up to 3 weeks post administration (9), and some clinical evidence suggests a protective role for IL-12 in psoriasis patients (34). Conversely, IL-23 is strongly associated with skin pathology. Injection of IL-23 is sufficient to induce psoriasis in mice that is partially dependent on TNF (35) and clinical trials have demonstrated that biologics against IL-23 are efficacious in patients with moderate to severe psoriasis (36, 37). In other models of dermatitis, LCs are thought to produce IL-23 to stimulate a pathogenic Th17 cell response in the skin (38–41). However, Th17 cells may only exacerbate disease in *Sharpin^cpdm^* animals in a modulatory capacity, as cpd is not dependent on T cells (27). Nonetheless, while it is conceivable that MIB2 limits an exacerbated Th17 response, we did not detect any MIB2-dependent changes in the Th17-associated cytokine, IL-17. More research is therefore needed to disentangle the role of these individual MIB2-regulated cytokines in the exacerbation of the cpdm phenotype.

Of note, in *Sharpin^cpdm^* mice, LCs drive the skin pathology by producing TNF (17). Specifically, both deletion of Sharpin and production of TNF within the LCs are required for this pathology, evidenced by experiments showing that *Sharpin^cpdm^* mice that have wild-type or TNF-deficient LCs do not develop dermatitis (17). These observations suggest that mutation of *Sharpin* in LCs may lead to the consequential production of TNF. In immune cells, TNF production is partially controlled by caspase-8 (42). Therefore, it is possible that by deleting *Sharpin*, increased caspase-8 activity exacerbates the production of TNF by the LCs without engaging cell death. Notably, what stimulates the production of TNF by LCs in the epidermis is unclear and it remains conceivable that loss of MIB2 may contribute to non-apoptotic caspase-8 signaling in these cells. Further to this, a role for CYLD in myeloid mononuclear phagocytes is implicated in the *Sharpin^cpdm^* dermatitis phenotype, although whether the function of CYLD is required in the LCs and/or infiltrating immune cells remains unclear (18). Considering that MIB2 negatively regulates CYLD (43, 44), disentangling how CYLD regulates the immune compartment *in vivo* may provide further insight into how MIB2 may limit the *Sharpin^cpdm^* phenotype.

In humans, the genetic perturbation of some regulators of TNF signaling, such as HOIP, NF-kappa B essential modulator (NEMO), or ovarian tumour domain deubiquitinase with linear linkage specificity (OTULIN), causes the presentation of inflammatory skin pathologies (45–49). These patient reports suggest that normal NF-κB signaling is an important regulator of skin homeostasis as is the case in mice. However, defined genetic perturbations in NF-kB signaling have not been clearly linked to the majority of dermatological skin diseases. MIB2 mutations have not been reported to cause inflammatory skin lesions, nor have they been identified in studies of human skin disease. Therefore, while it is conceivable that MIB2 limits the severity of the skin manifestations in the HOIP, NEMO, or OTULIN patients, it is likely otherwise dispensable for normal skin homeostasis. Future studies could explore the function of MIB2 in the HOIP-epidermal knock-out (EKO) (50, 51), OTULIN-EKO (52, 53), and NEMO^+/−^ (54, 55) mice, in the topical imiquimod model, or in the more recently described model of psoriasis caused by an autoactivating mutation in ras-related C3 botulinum toxin substrate 1 (RAC1), RAC1V12 (56).

Notably, our mass spectrometry analysis revealed a previously undiscovered facet of MIB2's function within the dermatological context of the Sharpin cpd mutation, demonstrating a significant enrichment in proteins associated with cellular division in the absence of MIB2. This unbiased observation may reflect the increased epidermal thickening we observe in the *Sharpin^cpdm^Mib2^−/−^* animals, a hallmark feature of the proliferative dermatitis phenotype. Whether the regulation of cell division by MIB2 is direct or specific to *Sharpin* deficiency remains to be determined, yet these results emphasize its involvement in regulating molecular processes beyond its established roles, underscoring the complexity of its functions in a disease context.

Overall, our study reveals a previously unknown role of MIB2 in the dermatological phenotype of the Sharpin cpd mutation. Previous studies have reported the upregulation of wound-healing molecules in cpdm mice, but their involvement in regulating the severity of the disease has remained unexplored. Our research establishes a clear link between the reduction of MIB2 and a significant decrease in the levels of several molecules involved in wound healing. While MIB2 mutations have not been linked to inflammatory skin lesions in humans, further investigation into the function of MIB2 in other genetic perturbations and skin disorders holds promise for gaining insights into skin homeostasis and related conditions.

## Materials and methods

### Animal handling

C57BL/6J mice were maintained in-house under specific pathogen-free conditions at the Walter and Eliza Hall Institute of Medical Research (WEHI), Australia. Animal rooms were maintained at approximately 21 ± 3°C at 40–70% humidity with a timed 14/10 h light dark cycle. Wild-type C57BL/6J mice were bred at WEHI and/or obtained from WEHI animal supplies (Kew, Australia). None of the mice used in our experiments had been previously used for other procedures. The animals presented with a healthy status and were selected independently of their gender for experimentation. Female and male mice were at least 6 weeks old at the time of experimentation. Wild-type and knock-out mice were cohoused from 14 days of age until 6–8 weeks when the experiment was initiated. All procedures for this study were approved by the WEHI Animal Ethics Committee, Australia. All research complied with all relevant ethical regulations for animal testing and research.

### Generation of mouse strains

The *Mib2^−/−^* mice were generated by the Melbourne Advanced Gene Editing Centre (MAGEC) laboratory (WEHI) on a C57BL/6J background. To generate *Mib2^−/−^* and *Mib2^KI/KI^* mice, 20 ng/μL of Cas9 mRNA, 10 ng/μL of sgRNAs (CGTGTGTCCTATGGTTAGCC and GTCCTGAGAACAAGGGCGTA), or (CGAAGATCTGAATGCGGTCG), respectively, were injected into the cytoplasm of fertilized one-cell stage embryos generated from wild-type C57BL/6J breeders. Twenty-four hours later, two-cell stage embryos were transferred into the uteri of pseudo-pregnant female mice. Viable offspring were genotyped by next-generation sequencing. Targeted animals were backcrossed twice to wild-type C57BL/6J to eliminate off-target mutations. *Sharpin^cpdm^* animals were originally generated and characterized on a C57BL/KaLawRij background (1) and have been previously backcrossed to the C57BL/6J background (13).

### Antibodies

The following primary antibodies were used for immunoblotting: anti-MIB2 (ProteinTech, 13696-1-AP, discontinued), antiactin conjugated to horseradish peroxidase (HRP; Santa Cruz, sc-47778), anticaspase-3 (Cell Signalling, 9662), anticleaved caspase-3 (Cell Signalling, 9661), antiprocaspase-8 (in-house, 3B10), anticleaved caspase-8 Asp387 (Cell Signalling; 9429, 8592), anti-RIPK1 (p-S166; Cell Signalling, 31122), anti-RIPK1 (BD Bioscience, 610459), anti-CYLD (p-S418; Cell Signalling, 4500S), anti-CYLD (Cell Signalling, 12797), anti-IκBα (p-S32/36; Cell Signalling, 9246), anti-IκBα (Cell Signalling, 9242), anti-p65 (p-S536; Cell Signalling, 8242), anti-p65 (Cell Signalling, 3033), anti-A20 (Cell Signalling, 4625), antitubulin (Cell Signalling, 2144), and GAPDH (Abnova Corporation, PAB6936). The following secondary antibodies were used for immunoblotting: peroxidase-affinipure rabbit antigoat IgG (H + L) (Jackson ImmunoResearch Labs, 305-035-003), peroxidase-affinipure goat antimouse IgG (H + L) (Jackson ImmunoResearch Labs, 115-035-003), peroxidase-affinipure goat antirabbit IgG (H + L) (Jackson ImmunoResearch Labs, 111-035-003), and peroxidase-affinipure goat antirat IgG (H + L) (Jackson ImmunoResearch Labs, 112-035-003). The following antibodies were used for immunohistochemistry: anticleaved caspase-3 (CST, 9661), anticleaved caspase-3 (R&D Systems, AF835), anti-F4/80 (CST, 70076), anti-CD3 (Agilent, A045201-2), anti-B220 (in-house, WEHI), anti-Ki67 (CST, 12202), and anti-CD45 (CST, 70257).

### Preparation of protein lysates from tissue samples

Tissues for analysis were dissected and frozen immediately on dry ice and stored at −80°C. Up to 100 mg of frozen tissue was sectioned off into a 2-ml snap-locked tube on ice. One tungsten metallic bead was added to each tube, followed by DISC lysis buffer supplemented with protease and phosphatase inhibitors (Tris–HCl [20 mM, pH 7.5], NaCl [2 mM], ethylenediaminetetraacetic acid [EDTA; 2 mM], Triton X-100 [1% v/v], glycerol [10% v/v], PhoSTOP phosphatase inhibitor [0.1 tablets/mL; Roche, 4906845001], and complete, EDTA-free protease inhibitor [0.02 tablets/mL; Roche, 4693132001], H_2_O) at a ratio of 10 μL/mg tissue. Tissues were disrupted using a TissueLyser II (30 Hz; Qiagen) for 1:30 min intervals until tissues were completely disrupted. The disrupted tissues were then incubated at 4°C for 30 min with vertical rotation to lyse the disrupted tissue. Samples were subsequently centrifuged at 15,000 rpm, 4°C to pellet nonlysed tissue and cellular debris. The lysates between the pellet and fat layer were extracted into new tubes and protein concentrations were assessed by the Pierce BCA assay (ThermoFisher, 23225) according to the manufacturer's instruction. For caspase activity measurements using the DEVDase assay, the complete, EDTA-free protease inhibitor was omitted from the lysis buffer, and skin lysates were assayed immediately after preparation.

### Preparation of mouse dermal fibroblasts

Tails from experimental animals were dissected and sterilized in 80% ethanol, then the skin was removed and incubated in complete keratinocyte serum-free media (KSFM) supplemented with dispase II (2.1 U/mL; Sigma-Aldrich, D4693) at 37°C for 2 h with agitation. Next, the epidermis and dermis were separated using sterilized tweezers, the dermis was cut into small ∼50 mm sections and incubated in 3 mL of Hanks' balanced salt solution (HBSS) with calcium and magnesium (ThermoFisher, 14025092) supplemented with Liberase TM (0.13 Wünsch U/mL; Sigma-Aldrich, 5401119001) for 2 h at 37°C with agitation. The softened dermis was mashed through a 100-μm cell strainer using a plastic syringe plunger into 10 mL DMEM containing FBS. Cells were centrifuged at 1,500 G for 5 min, then resuspended in 10 mL DMEM containing FBS and seeded onto 10 cm tissue culture treated plates and cultured at 37° C, 10% CO_2_.

For experimentation, cells were seeded in 96-well tissue culture treated plates for IncuCyte analysis or 24-well tissue culture treated plates for western blot analysis. Cells were seeded at equal densities so that by experimental endpoint, the final density would be 100% (e.g. cells were seeded at 25% density for a 48-h time course, ∼2 division cycles). For IncuCyte analysis, cell medium was replaced with fresh medium containing propidium iodide (PI, 1 μg/mL; Sigma-Aldrich, P4170) with IDN-6556 (10 μM, Cayman Chemical), Nec1s (10 μM,), GSK’872 (5 μM, Merck; 5303890001) or the Smac mimetic, compound A (1 μM, TetraLogic Pharmaceuticals), or DMSO vehicle 15 min before recombinant murine TNF (200 μg/mL, PreproTech; 315-01A) stimulation. Post-TNF stimulation, cells were immediately placed in the IncuCyte to be imaged every 2 h for 24–48 h. Each treatment was assayed in duplicate, with four images taken per well.

### Caspase activity measurements using the DEVDase assay

Fifty micrograms of purified protein lysate from the dorsal thoracic skin were aliquoted into two wells of a 96-well plate, with either IDN-6556 (20 μM final concentration) or an equivalent volume of DMSO vehicle. Samples were incubated at room temperature for 30 min with shaking. AMC-DEVD-AMC reagent (BD Biosciences, cat #: 556449) was prepared according to manufacturer instruction, and 200 μL of working solution (20 μM) was added to each well. The plate was shielded from light and incubated for 16 h with shaking then fluorescence intensity was measured on a CLARIOstar Plus microplate reader (BMG Labtech) with an excitation of 380–8 nm and emission of 445–30 nm.

### Cytokine measurements from plasma and skin

Peripheral blood was centrifuged at 500 G for 5 min and plasma was extracted into a new tube and immediately frozen at −80°C and thawed on ice immediately prior to analysis. Plasma cytokines and chemokines were measured using the Bio-Plex pro mouse cytokine 23-plex assay kit (Bio-Rad, M60009RDPD). Plasma was diluted 1:4 in assay diluent or skin tissue lysate was diluted to 20 μg/sample in assay diluent to an equal volume along with serially diluted standards. Samples were added to a plate containing magnetic antibody-coupled beads for each analyte and incubated at room temperature for 30 min with rotation (300 rpm). The plate was then washed with the Bio-Plex washing buffer and secondary antibodies were added to the plate and incubated at room temperature for 30 min with rotation (300 rpm) followed by additional washing. Streptavidin-Phycoerythrin (PE) was added to the plate and incubated at room temperature for 10 min with rotation (300 rpm) in the dark. Assay buffer was added to each well, and the plate was analyzed on the Bio-Plex 200 machine. Plasma analyte concentrations were interpolated from a standard curve of fluorescence intensity. Values below the lowest standard of the curve were assigned the value of the lowest standard.

### Preparation of cell lysates for sodium dodecyl sulfate–polyacrylamide gel electrophoresis

For MDFs at the experimental endpoint, the cell supernatant was aspirated and cells were lysed in 1× in DISC lysis buffer supplemented with protease and phosphatase inhibitors. Protein concentrations were determined using the Pierce BCA assay (ThermoFisher, 23225) according to the manufacturer's instruction and assayed in duplicate. NuPAGE lithium dodecyl sufate (LDS) lysis buffer (Invitrogen) containing freshly added β-mercaptoethanol was diluted to working concentrations (1× for LDS lysis buffer, 143 mM for β-mercaptoethanol) in the remaining protein lysates and heated at 70°C for 10 min. Samples were loaded onto sodium dodecyl sulfate polyacrylamide gel electrophoresis (SDS–PAGE) gels at volumes dictated by the BCA results to achieve equal protein concentrations.

### SDS–PAGE and immunoblotting

Proteins were separated by SDS–PAGE on 4–12% gradient gels (Invitrogen) and transferred onto immoblon-E polyvinyl difluoride membranes (Merck). Protein loading accuracy was assessed by staining the membranes for 5 min with Ponceau stain. Membranes were blocked with tris-buffered saline with tween-20 (TBS-T)-containing skim milk (5% w/v) for 20 min prior to immunoblotting with primary antibodies overnight at 4°C. All primary antibodies were diluted at 1:1,000 in TBS-T containing bovine serum albumin (BSA, 5% w/v; Sigma-Aldrich A8022) and sodium azide (0.04% (w/v)). Relevant HRP-conjugated secondary antibodies (Jackson ImmunoResearch Labs) were diluted 1:10,000 in TBS-T containing skim milk (5% w/v) and applied for 1 h at room temperature. Membranes were subjected to 3×5 min washes in TBS-T between antibody incubations and 4×5 min washes after secondary antibody incubation. Enhanced chemiluminescence (ECL) development (Millipore, Bio-Rad) was performed prior to protein detection using the ChemiDoc touch imaging system (Bio-Rad). Where a membrane was to be probed with another primary antibody, the membrane was subjected to 1 × 1 h wash in TBS-T containing sodium azide (0.2% w/v), then 1 × 5 min wash in TBS-T prior to overnight incubation with the sequential primary antibody.

### Mass-spectrometry-based proteomics

Twenty micrograms of protein lysates were subjected to Suspension TRAPping (S-TRAP) sample digestion method (Protifi, USA) with the following exceptions. Samples were reduced (10 mM tris[2-carboxyethyl]phosphine [TCEP]) and alkylated (20 mM chloroacetamide) at 55°C for 15 min. Protein samples were loaded onto micro S-traps and were washed three times with 90% methanol and 100 mM triethylammonium bicarbonate (TEAB), followed by an additional three washes with 50% dichloromethane/50% methanol to minimize fat and other hydrophobic contaminants present in the tissue lysates. Following trypsin/Lys-C digestion peptides were eluted, lyophilized to dryness using CentriVap (Labconco), and a final peptide clean up performed using SDB stage tips (GL Sciences). Peptide samples were reconstituted in 0.1% FA/2% ACN and were separated by reverse-phase chromatography (IonOpticks Aurora 75 μm ID, OD 360 μm × 15 cm length, 1.6 μm C18 beads) using a custom nano-flow HPLC system (Thermo Ultimate 300 RSLC Nano-LC, PAL systems CTC autosampler). The HPLC was coupled to a timsTOF Pro (Bruker) equipped with a CaptiveSpray source. Peptides were loaded directly onto the column at a constant flow rate of 400 nL/min with buffer A (99.9% Milli-Q water, 0.1% FA) and eluted with a 30-min linear gradient from 2 to 34% buffer B (90% ACN, 0.1% FA). The timsTOF Pro (Bruker) was operated in diaPASEF mode using Compass Hystar 5.1. The settings on the thermal ionization mass spectrometry (TIMS) analyzer were as follows: Lock Duty Cycle to 100% with equal accumulation and ramp times of 100 ms, and 1/K0 start 0.6 V·s/cm^2^ end 1.6 V·s/cm^2^, capillary voltage 1,400 V, Dry Gas 3 L/min, dry temp 180°C. diaPASEF acquisition was performed using methods previously described (57). Briefly, 16× 25 *m*/*z* precursor isolation scans (resulting in 32 windows) were aligned across the *m*/*z* (400–1,200) and ion mobility (0.8–1.4), with 1 Da overlap, and CID collision energy ramped stepwise from 20 eV at 0.8 V·s/cm^2^ to 59 eV at 1.3 Vs/cm^2^.

### Data processing and statistical analysis for mass-spectrometry-based proteomics

DIA data were analyzed using DIA-NN 1.8 in library-free mode (58). diaPASEF d. files were searched against reviewed sequences from mouse Uniprot Reference Proteome (downloaded November 2022) with the following settings: trypsin specificity, peptide length of 7–30 residues, cysteine carbidomethylation as a fixed modification, variable modifications set to n-terminal protein acetylation and oxidation of methionine, the maximum number of missed cleavages at 2. Mass accuracy was set to 10 ppm for both MS1 and MS2 spectra and match between runs (MBR) was enabled, and filtering outputs set at a precursor *q*-value <1%.

Data processing and analysis were conducted using R (version 4.2.1). Proteins lacking proteotypic precursors, with a *q*-value >0.01, and/or identified by fewer than two peptides were excluded. Additionally, only proteins quantified in at least 50% of replicates in at least one condition were retained, resulting in a total of 3,439 proteins for further analysis. Subsequently, protein intensities were log_2_ transformed. A distinction was made between whether missing values were missing at random (MAR) or missing not at random (MNAR). Since the missingness was identified as MNAR, the Barycenter method, implemented in the msImpute package (v. 1.9.0), was applied. The dataset was then normalized using the cyclicloess method, as implemented in limma (v. 3.52.4). Differential analysis was conducted using limma. A protein was deemed significantly differentially expressed if the false discovery rate (FDR) was ≤5% after correction for multiple testing using the Benjamini–Hochberg (BH) approach.

### Hematology

Peripheral blood from animals was extracted either by eye-bleed or cardiac bleed when animals had reached experimental endpoint into EDTA-lined tubes (Sarstedt) and analyzed by an ADVIA 2120i hematology system (Siemens). In cases where plasma was extracted, plasma was replaced with an equal volume of phosphate-buffered saline (PBS) before ADVIA analysis.

### Histopathology and immunohistochemistry

Animals were either humanely euthanized or sent to the APN Histopathology and Organ Pathology Service at the University of Melbourne for necropsy. Tissues from humanely euthanized animals were fixed in 10% neutral buffered formalin for 24–48 h, then embedded in paraffin, sectioned, and stained with hematoxylin and eosin (H&E), CC3, F4/80, CD3, B220, Ki67, or CD45. Histological quantification of epidermal thickness and CC3 positivity of the thoracic dorsal skin was performed in QuPath. For epidermal thickness, at least 10 measures per section were made at equal intervals over at least two sections, with the average of all measures per animal plotted. For determination of CC3 positivity, positively stained cells in the epidermis were marked by the QuPath software in an analysis mask made around the epidermis with an area of 1.0 ± 0.4 mm^2^. Total cell numbers were determined based on the hematoxylin stain. Genotypes were blinded for all histological quantifications.

### PCR-based genotyping

Tail or ear clips from *Mib2^−/−^*, *Mib2^KI/KI^*, *Sharpin^cpdm^*, *Sharpin^cpdm^Mib2^−/−^*, or *Sharpin^cpdm^Mib2^KI/KI^* animals were taken at weaning (3 weeks) and digested in 100 μL DirectPCR Tail lysis buffer (mouse tail; Viagen Biotech) supplemented with proteinase K (1:1,000, Sigma-Aldrich P4850) at 55°C for 5 h or overnight with agitation. Tail lysates were heat inactivated at 95°C for 30–45 min. Genotyping PCR was performed in a 14-μL reaction mixture containing 1.5 μL sample lysate and 12.5 μL PCR master mix comprising of GoTaq Green mixture (1×; Promega M7123), forward and reverse primers (500 nM each for *Mib2* alleles, 1 μM each for *Sharpin* alleles). DNA bands were separated on a 1.5% (w/v) agarose gel containing SYBR Safe DNA gel stain (1:33,333; ThermoFisher S33102) and detected using a ChemiDoc imaging system (Bio-Rad, 17001401). All images were processed using the Image Lab software.

### Genotyping primers

Wild-type (*Mib2*Δex1–6 control):

F: CCACATGGGCATCTACAATG

R: TGAGGGGGTTGTTCAAAGAC

*Mib2*Δex1–6:

F: TGCTGATGTTCTTGCTTCTT

R: TCCACACAGCTTCCACAGAC

Wild-type (*Mib2^F920A^* control):

F: GCGACCGCATTCAGATCTTC

R: TTGTGTCAAACCAAAGCCAG

*Mib2^F920A^*:

F: GCGACCGCATTCAGATCGCC

R: TTGTGTCAAACCAAAGCCAG

Wild-type (*Sharpin^cdpm^* control):

F: TTAGGCACCGAGCCTGGGG

R: CTAAAGCGCATGCTCCAGACTGCCTTG

*Sharpin^cdpm^*:

F: TTAGGCACCGAGCCTGGGC

R: CTAAAGCGCATGCTCCAGACTGCCTTG

### Clinical scoring of cpd

Scoring of *Sharpin^cdpm/cdpm^Mib2^−/−^* and *Sharpin^cdpm/cdpm^Mib2^KI/KI^* was performed in a blinded fashion by animal technicians familiar with the phenotype and the scoring system to minimize bias. Littermate *Sharpin^cdpm/cdpm^* control mice were scored alongside experimental *Mib2* mutant subjects. Clinical grading of the dermatitis was performed on an ordinal 5-point scale (0–4), based on the developed scoring system published previously (17). Severe dermatitis free curves measure the percentage of assessed animals that have not reached clinical score of 4 (endpoint) at a given age. Several animals required euthanasia due to ear infections (irrespective of genotype) prior to developing severe dermatitis and were excluded from all analyses.

## Supplementary Material

pgad438_Supplementary_Data

## Data Availability

Reagents generated during the current study are available from the corresponding authors upon reasonable request. All relevant data and supplementary materials supporting the findings of this study are included in the manuscript or in supplementary materials. Mass spectrometry proteomics data were deposited in the ProteomeXchange Consortium via the PRIDE partner repository with the dataset identifier PXD045850 (username: reviewer_pxd045850@ebi.ac.uk; password: 5aVt7EGK).
