# Supplementary material for: Mind bomb 2 limits inflammatory dermatitis in Sharpin mutant mice independently of cell death
Source: PNAS Nexus. 2023 Dec 18;3(1):pgad438. doi: 10.1093/pnasnexus/pgad438 (PMC10753164; doi:10.1093/pnasnexus/pgad438)
Supplement: pgad438_Supplementary_Data [file pgad438_supplementary_data.docx]

**Supporting Information for**

Mind Bomb 2 limits inflammatory dermatitis in *Sharpin* mutant mice independently of cell death

Daniel S Simpson, Holly Anderton, Jumana Yousef, Vineet Vaibhav, Simon A Cobbold, Esther Bandala-Sanchez, Andrew J Kueh, Laura F Dagley, Marco J Herold, John Silke, James E Vince and Rebecca Feltham

Rebecca Feltham and James E Vince

Email: [feltham.r@wehi.edu.au](mailto:feltham.r@wehi.edu.au), [vince@wehi.edu.au](mailto:vince@wehi.edu.au)

**This PDF file includes:**

Figures S1 to S5

**Figure S1** **
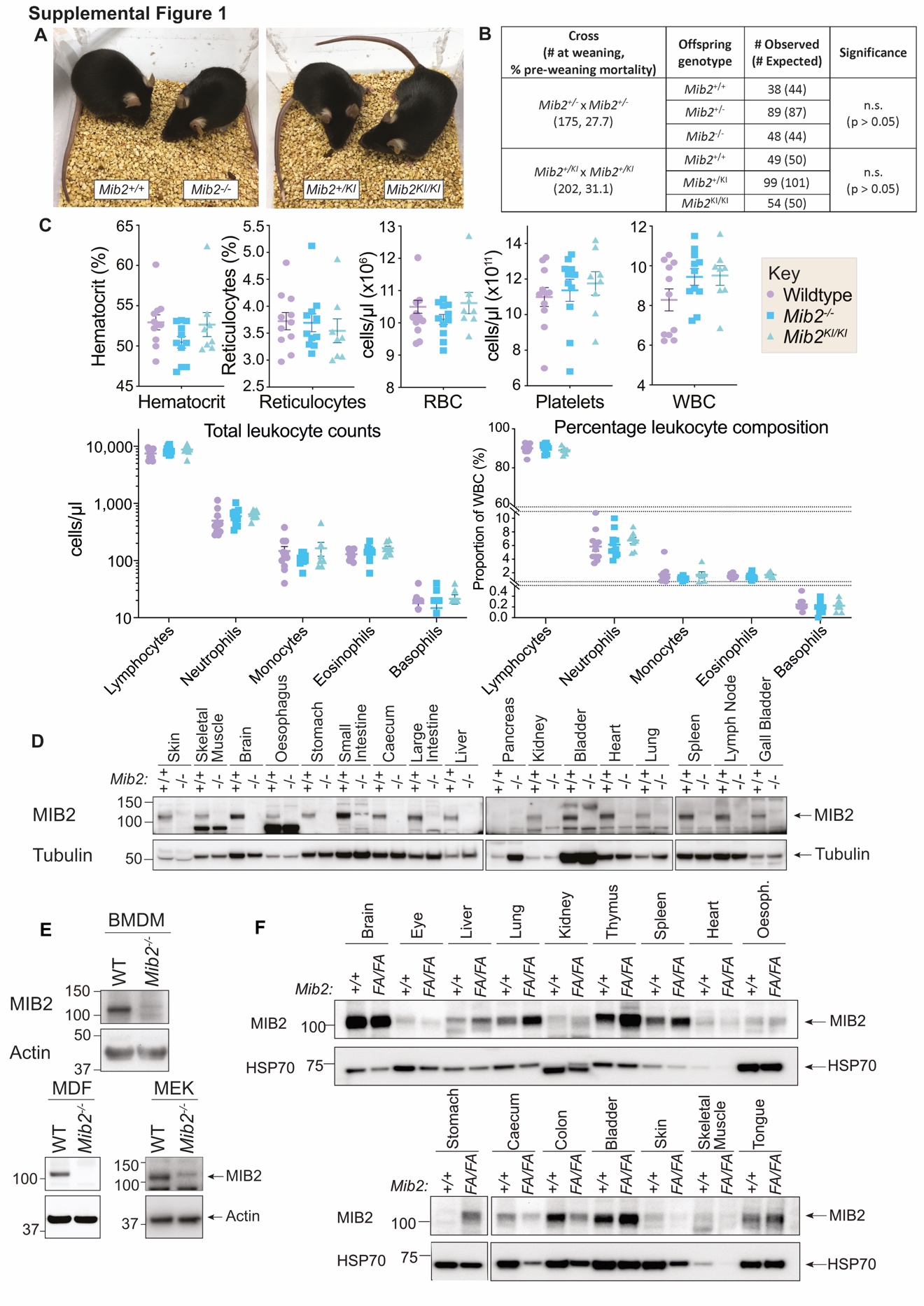
**

*(Legend on next page)*

**Figure S1. Deletion or inactivation of MIB2 causes no overt phenotype in mice**A. Images of adult *Mib2^-/-^* and *Mib2^KI/KI^* animals alongside litter-mate wild-type and *Mib2^KI/+^* control animals.
B. Observed *versus* expected table of offspring from heterozygous crosses of *Mib2* mutant colonies. n.s = not significant as determined using a Chi-squared test.
C. ADVIA analysis of whole blood samples from 9 to 11-week-old wild-type (n = 11), *Mib2^-/-^* (n = 11), and *Mib2^KI/KI^* (n = 8) animals. Percentage hematocrit, red blood cell (RBC), reticulocytes, platelet, and white blood cell (WBC) counts are shown. Leukocyte profiles detailing total and percentage composition of the total WBC are shown. Datapoints represent independent biological replicates. Mean ± SEM. All comparisons were non-significant as determined using the Kruskal-Wallis test with Dunn’s correction for multiple comparisons.
D. Western blot analysis of tissues from wild-type or *Mib2^-/-^* animals. Representative western blot of n = 3 independent experiments.
E. Western blot analysis of bone marrow derived macrophages (BMDM), mouse dermal fibroblasts (MDF) and murine epidermal keratinocytes (MEK) isolated from wild- type and *Mib2^-/-^*animals. Representative western blot of n = 3 independent experiments.

F. Western blot analysis of tissues from wild-type or *Mib2^F920A/F920A^ (FA/FA, Mib2^KI/KI^)* animals.

Figure S2

 Figure S2. MIB2 does not impact on immune cell infiltration or epidermal proliferation in *Sharpin^cpdm^* mice.
A. Pre-weaning mortality rates for the indicated breeding pairs.
B. Observed versus expected table of offspring from heterozygous crosses of *Sharpin* mutant colonies with either homozygous or heterozygous deletion of *Mib2*. n.s = not significant as determined using a Chi-squared test.
C. Histopathology results for 8 – 9 week old animals of the indicated genotypes. N.Obs = No noted pathology observed, Y = Pathology is observed. Each column represents an independent biological replicate. n = 3 - 5.
D. IHC staining of dorsal thoracic skin sections from 8 – 9 week old animals depicting a Ki67 (HRP Magenta) and CD3 (DAB) duo-stain, CD45 (DAB) stain and F4/80 (DAB) stain. n = 3 biological replicates per group. Images are representative of independent biological replicates. Scale bar = 200 μm.

Figure S3

**Figure S3. Skin-independent phenotypes of *Sharpin^cpdm^* animals are not impacted by the deletion of MIB2**A. F4/80, CD3, B220, Cleaved caspase-3 (CC3) (Cell Signalling Technologies Antibody (CST), and RnD Systems (R&D) antibody) staining of liver and spleen sections from the indicated genotypes. n = 2 – 5. Images are representative of independent biological replicates. Scale bar = 100 μm (liver and spleen CC3), 500 μm (spleen F4/80, CD3 and B220).
B. ADVIA analysis of whole blood samples from 9 to 18-week-old animals of the indicated genotypes. Percentage hematocrit, platelet counts, and leukocyte profiles detailing percentage composition of the total WBC are shown. Datapoints represent independent biological replicates. Mean ± SEM. All comparisons were non- significant as determined using the Mann-Whitney U test.

Figure S4

*(Legend on next page)*

**Figure S4. MIB2 does not impact TNF-mediated cell death in murine dermal fibroblasts from *Sharpin^cpdm^* mice**
A. Cell death measured by IncuCyte tracking of propidium iodide (PI) uptake of primary MDFs stimulated with; FLAG-TNF (100 ng/ml), IDN-6556 (10 μM), Nec1s (10 μM), GSK’872 (5 μM) or the Smac mimetic, Compound A (Cp. A, 1 μM) for 24 hours for the indicated genotypes.
B. Representative experiment (from 2 independent repeats) containing cells from 3 independent mice. Error bars represent the mean of biological replicates ± SEM. All comparisons between *Sharpin^cpdm^Mib2^-/-^* and *Sharpin^cpdm^Mib2^+/+^* were non-significant as determined using a Friedman test with Dunn’s correction for multiple comparisons.
C, IncuCyte images of cells at 24 hours post treatment with a confluence mask (white lines) overlayed and PI positive cells (red) shown. Analysis of these images was used to obtain PI+ count/mm2 cell area in (A). Scale bar = 400 μm.
D. Western blot analysis of FLAG-TNF (100 ng/ml) treated MDFs at the indicated timepoints for the indicated genotypes. Representative western blot of n = 2 independent experiments.

Figure S5

** Figure S5. Plasma cytokine levels in MIB2 deleted mice**Heat map visualising cytokine and chemokine concentrations (pg/ml) measured by a multiplex assay of plasma from animals of the indicated genotypes at 4 weeks old and subsequently at a clinical dermatitis score of 4 (or equivalent age for *Sharpin^+/+^* animals). n = 9 independent biological replicates. Significance between the indicated genotypes were determined multiple Mann-Whitney U tests with a Holm*-*Šídák correction for multiple comparisons. Significant comparisons are denoted as Adj. p-value: < 0.05 (*), ≤ 0.01 (**), ≤ 0.001 (***).

Figure S6

**
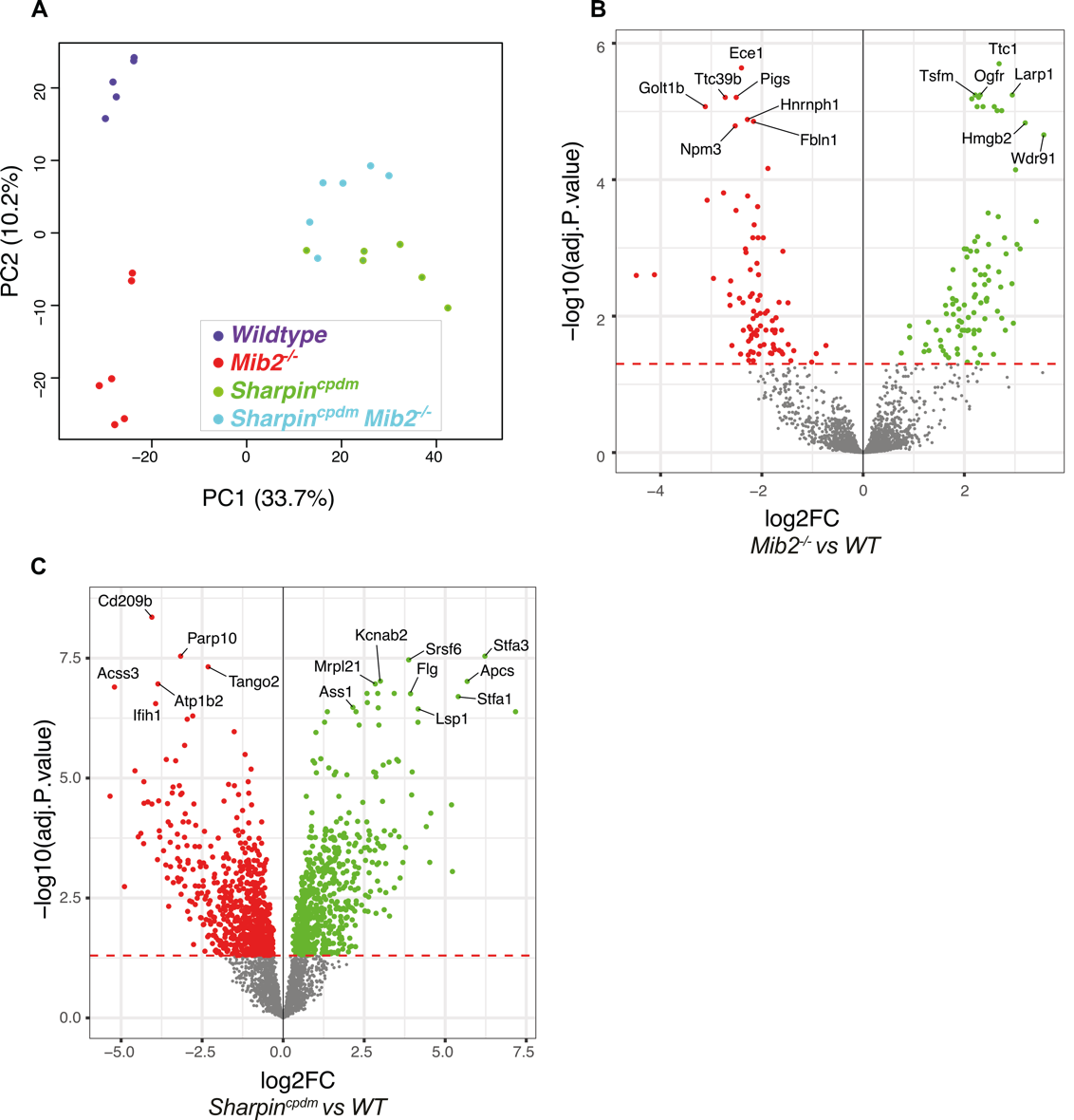
**

**Figure S6. Comparative mass spectrometry analysis of protein expression in skin lysates reveals differential protein expression in the absence of MIB2**

A. Principle component analysis plot of 3,439 of the most variable proteins across the four genotypes, including wildtype (n =5), *Mib2^-/-^* (n =6), *Sharpin^cpdm^* (n =6) and *Sharpin^cpdm^Mib2^-/-^* (n =6). The plot shows the separation of samples based on differential principal components (PCs).

B. Volcano plot illustrating proteins exhibiting significant differential expression. The –log10 (Benjamini-Hochberg corrected *P* value) is plotted against the log_2_ protein fold changes comparing *Mib2^-/-^* (green) vs wildtype (red). Green = proteins upregulated in *Mib2^-/-^* mice, and red = proteins upregulated in wildtype mice. Proteins were deemed differentially regulated with an adjusted *p*-value of ≤0.05 (proteins coloured in red and green).

C. Volcano plot illustrating proteins exhibiting significant differential expression. The –log10 (Benjamini-Hochberg corrected *P* value) is plotted against the log_2_ protein fold changes comparing *Sharpin^cpdm^* (green) vs wildtype (red). Green = proteins upregulated in *Sharpin^cpdm^* mice, and red = proteins upregulated in wildtype mice. Proteins were deemed differentially regulated with an adjusted *p*-value of ≤0.05 (proteins coloured in red and green).
